# Improving the reliability of underwater gait analysis using wearable pressure and inertial sensors

**DOI:** 10.1371/journal.pone.0300100

**Published:** 2024-03-21

**Authors:** Cecilia Monoli, Manuela Galli, Jeffrey A. Tuhtan

**Affiliations:** 1 Department of Computer Systems, Tallinn University of Technology, Tallinn, Estonia; 2 Department of Electronics, Information and Bioengineering, Politecnico di Milano, Milano, Italy; Polytechnic University of Marche: Universita Politecnica delle Marche, ITALY

## Abstract

This work addresses the lack of reliable wearable methods to assess walking gaits in underwater environments by evaluating the lateral hydrodynamic pressure exerted on lower limbs. Sixteen healthy adults were outfitted with waterproof wearable inertial and pressure sensors. Gait analysis was conducted on land in a motion analysis laboratory using an optoelectronic system as reference, and subsequently underwater in a rehabilitation swimming pool. Differences between the normalized land and underwater gaits were evaluated using temporal gait parameters, knee joint angles and the total water pressure on the lower limbs. The proposed method was validated against the optoelectronic system on land; gait events were identified with low bias (0.01s) using Bland-Altman plots for the stride time, and an acceptable error was observed when estimating the knee angle (10.96° RMSE, Bland-Altman bias -2.94°). The kinematic differences between the land and underwater environments were quantified, where it was observed that the temporal parameters increased by more than a factor of two underwater (p<0.001). The subdivision of swing and stance phases remained consistent between land and water trials. A higher variability of the knee angle was observed in water (CV = 60.75%) as compared to land (CV = 31.02%). The intra-subject variability of the hydrodynamic pressure on the foot (${\text{CV}}_{z}^{\text{foot}}$ = 39.65%) was found to be substantially lower than that of the knee angle (CV_z_ = 67.69%). The major finding of this work is that the hydrodynamic pressure on the lower limbs may offer a new and more reliable parameter for underwater motion analysis as it provided a reduced intra-subject variability as compared to conventional gait parameters applied in land-based studies.

## Introduction

Physical activity in water can reduce the risk of injury during rehabilitation exercises due to the lessening of joint loading caused by buoyancy and the increased resistance to motion caused by drag [1]. Water rehabilitation can also aid in the management of chronic conditions [2], promote injury recovery, enhance exercise performance [3], and has shown to positively contribute to the psychological well-being of participants [4].

The authors’ recent systematic review highlighted the scarcity of quantitative underwater motion analysis methods [5]. The methods and protocols used in underwater studies are commonly taken from land-based methods [6], but may be unsuitable for aquatic environments [7]. Optoelectronic systems remain the gold standard for land-based motion analysis, but rely on cable-connected infrared cameras and passive light-reflective markers [8]. Accordingly, optoelectronic systems are not well-suited for both outdoor and underwater applications [9]. Previous underwater studies have made use of external cameras to monitoring motion in a underwater treadmill tank [10], and a similar system has been developed which utilized blue LEDs and cameras for aquatic motion analysis [11]. Waterproof action cameras have also been used for kinematic analysis [9], where the analysis was restrained to planar investigations with a limited field of view [12].

Wearable inertial measurement units (IMUs) can be implemented as an alternative to optoelectronic and camera-based motion analysis systems for land-based and underwater motion analysis [13]. Unlike optoelectronic and camera-based methods, IMUs do not have limitations on the field of acquisition and can capture physical activities in natural and highly unstructured settings [14]. Previous studies have performed underwater motion analysis using IMUs to assess the walking gait [15–18], gait initiation [19, 20] as well as double and single limb squat exercises [21]. Although the physical activities considered in these works was often similar, the protocols adopted differed substantially between studies. This variability indicates an overall lack of structured clinical procedures and methodologies for underwater motion analysis. These shortcomings hinder the cross-comparison of findings and effective quantitative kinematic analysis, limiting the understanding of underwater characteristics of motion [5].

This paper illustrates that IMUs outfitted with pressure sensors, (PIMU) can provide a new and reliable method for motion analysis of the walking gait in water. The choice of walking in this study stemmed from its predictable patterns and extensive application in rehabilitation [22]. Gait analysis is an established method for assessing locomotion, diagnosis and the general well-being of test subjects [23–25]. The objectives of this work are three-fold: (i) to compare PIMU performance against an optoelectronic system as a land-based reference, (ii) to quantify the kinematic differences between land and underwater gaits, and (iii) to evaluate the reliability of the lateral hydrodynamic pressure on the lower limbs as a new motion analysis parameter for underwater gaits. To achieve the first two objectives, temporal gait parameters and knee angle kinematics were analyzed on land and underwater. Finally, the hydrodynamic pressure time series during a gait cycle at the thigh, shank and foot were compared with that of the knee angle.

## Materials and methods

### Participants

Sixteen adults were enrolled in this study: 9 Females: 24.8±1.1 years, 1.66±0.06 m height, 59.2±6.7 kg mass and 7 Males: 25.4±2.9 years, 1.78±0.05 m height, 73.6±11.6 kg mass. All subjects were healthy with no functional impairments, neurological or orthopedic conditions and were free of musculoskeletal injury or pain at the time of data collection. Additionally, the volunteers had no previous experience with water rehabilitation exercises. The experimental tests on land and underwater were conducted in compliance with the World Medical Association Declaration of Helsinki and were approved by the Ethics Committee of Politecnico di Milano (Decision 22/2021 on June 14th, 2021. Milan, Italy). The recruitment of volunteers started on September 1st, 2021 and was completed on December 20th, 2021. Written informed consent of the participants was collected.

### Inertial and pressure wearables

**Fig 1:**
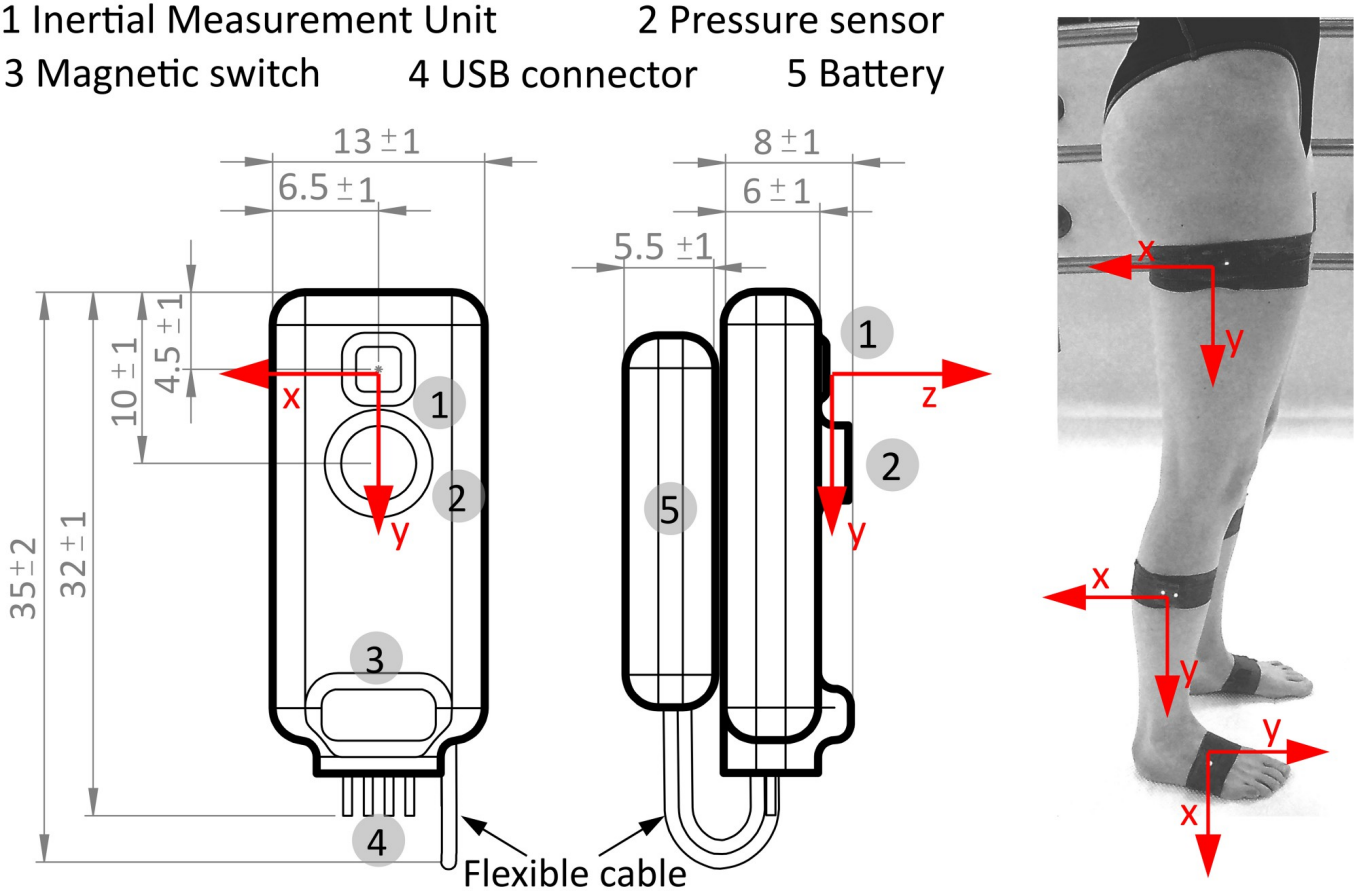
Technical drawing and placement of PIMU sensors for land and underwater walking gait motion analysis. (Left) Dimensions (mm) and locations of the main components. (Right) Placement and axial orientations of three PIMU on a test subject. The measurement axes of each sensor are highlighted in red.

This study implemented Pressure and Inertial Measurement Units (PIMU) sensors designed at the Tallinn University of Technology (Tallinn, Estonia) [26] for underwater kinematics. The wearable loggers are ideally suited for human motion analysis because they are small and lightweight (6.9 g dry mass), minimizing potential discomfort and interference with the subjects’ movement. The PIMU sensors log data at 100 Hz and include a tri-axial IMU (BMX160, Bosch Sensortec, Germany) which incorporates a linear accelerometer, gyroscope and magnetometer as well as a pressure sensor (MS5837–2BA, TE Connectivity, Switzerland). The devices are wirelessly activated using a magnetic switch after placement on the test subjects. The data are stored as comma-separated values (CSV) to an onboard memory module with 2 GB storage, and are retrieved through a USB connection by downloading the CSV file after each activity. Table 1 summarizes the technical characteristics of the loggers, and Fig 1 provides the dimensions and placement of the main components.

**Table 1:** Technical characteristics of the wearable, waterproof Pressure and Inertial Measurement Unit (PIMU).

Dimensions	35x13x13.5 mm (length x height x width)
Mass in air	6.9 ± 0.3 g
Microcontroller	32-bit ARM Cortex-M0+ SAM D21G
Absolute orientation sensor	BMX160 (Bosch)
Pressure sensor	MS5837–02BA (TE Connectivity)
Battery	Lithium-Polymer(+4v with USB charging) 3.7V 40mAh
Data Storage	SD card, 2 GB
Data logging rate	100 Hz
Waterproofing	Epoxy resin

### Experimental protocol

All subjects participated in two distinct trials: first, a land-based trial and afterwards the underwater trial. In both trials, subjects were asked to walk ten times in a straight line, at their preferred self-selected speed, with their arms folded across their chest. Three PIMUs were positioned laterally on the right leg, as shown in the right panel of Fig 1. The sensors were fixed with self-adhesive medical tape at the approximate center of mass of thigh, shank and foot [27], following a modified version of the Outwalk protocol [28].

The land trial fully addressed the first objective, which was to validate the PIMU motion analysis data through comparison with the optoelectronic system. In addition, the land trial provided data needed for the second objective, to evaluate differences between land and underwater gaits. The trial was conducted in the Posture and Movement Analysis Laboratory “Luigi Divieti” of Politecnico di Milano (Milan, Italy). The walking gait was recorded simultaneously with the three PIMU placed on the right lower limb and an 8-camera optoelectronic system BTS-SmartDX 400 (BTS Bioengineering S.p.a., Italy) sampling at 100Hz. The optoelectronic markers were placed on the test subjects following the Davis protocol [29]. To synchronize the optoelectronic system and the sensors, a two-pose static calibration after [30] was performed before each trial. Subjects were asked to stand upright (pose one) and then lift their right leg, in hip flexion with the knee flexed to a comfortable angle (pose two) for at least 2 seconds. After the completion of the calibration poses, the volunteers initiated walking, beginning with their right leg.

The underwater trial was conducted to fulfill the second and third objectives, namely to investigate the differences between land and underwater gaits and evaluate the hydrodynamic pressure as a potentially new parameter for underwater motion analysis. The modified Outwalk protocol used in the land-based trials was repeated in water, with the three PIMUs placed on each subject’s right lower limb. The underwater trials were conducted at the rehabilitative swimming pool of the Enjoy Sport Center (Cernusco sul Naviglio, Milan, Italy). The pool has a fixed depth of 1.20m, is 3m long, and the water temperature remained at 31°C throughout the trials. Following the protocol of the land-based trial, the PIMU calibration poses and gait initiation with the right leg were performed in water to ensure consistency between trials.

### Data processing and analysis

The second right-leg stride temporal gait parameters and knee angular kinematics were assessed for land and water trials and the hydrodynamic pressure was considered for the water trial. All parameters were normalized over the gait cycle to enable cross-comparison between repetitions and subjects. A total of 160 samples per parameter were evaluated: 16 subjects with 10 repetitions.

The standard gait classification was applied, defining a gait cycle as the motor tasks between two subsequent ipsilateral heel strikes [24]. The toe-off is the moment of final contact, occurring between two heel strikes and is the other main gait event. The identification of these three gait events, two heel strikes and the toe-off occurring in between, enabled to estimate the temporal parameters of interest. The first is the stride time, which is the duration of a complete gait cycle. The second is the stance time, which is the fraction of the gait cycle when the foot is on the ground and the third is the swing time, corresponding to the remaining fraction of the gait when the foot is lifted from the ground and moves forward. The equations used to evaluate the temporal parameters are reported in the supporting information S1 Appendix. The stance and swing fractions were expressed also as a percentage of the normalized gait cycle for the comparison between land and water trials. The knee joint kinematic in the sagittal plane was investigated normalizing the flexion-extension angle over the gait cycle. In addition to the angular kinematics, the maximal flexion and maximal extension were identified and the Range Of Motion (ROM) was estimated as the difference between them.

The optoelectronic data was processed using the BTS Smart Clinic software (BTS Bioengineering S.p.a., Italy) by manually identifying the gait events. Data collected with the PIMU sensors was post-processed in MATLAB (version R2022b, Mathworks Inc., USA). A bespoke algorithm was developed by the authors to automatically detect gait events based on the acceleration magnitude of the foot mounted sensor. The PIMU knee angle was estimated from the gyroscope and accelerometer data after applying the Madgwick filter to calculate the relative angle between the thigh and shank mounted IMUs, following Song et al. [31]. The hydrodynamic pressure was processed by subtracting the median value for each dataset to account for the different heights of the subjects, and was subsequently normalized in time over the gait cycle. Hydrodynamic pressure parameters similar to the knee angle were estimated: maximal and minimal pressure as well as the Range Of Pressure (ROP) which was calculated as their difference.

Addressing the first objective of this investigation, the PIMU-based method was validated by comparing its performance with the optoelectronic system. For temporal and joint parameters the root mean squared error (RMSE), Spearman correlation coefficient (*ρ*) and Bland-Altman plots were evaluated to assess measurement errors, biases and reliability. Differences between the land and water trials were assessed comparing descriptive statistics of the temporal parameters and joint kinematics, to address the second objective. The Brunner-Munzel test using a 95% confidence interval was performed [32, 33] for pair-wise comparison of the PIMU gait parameters on land and underwater and between the PIMU and optoelectric systems on land. This non-parametric test method was chosen as it is a generalised and more robust version of the Mann-Whitney U test which does not require the assumption of equal variances between sample populations [34]. The test groups were generated based on the ensemble gait data from all participants for each system (PIMU vs. optoeletronic) and environment (land or underwater).

Lastly, the third objective evaluated the lateral hydrodynamic pressure parameter as a novel underwater motion analysis parameters estimating its coefficient of variation (CV). The CV was based on the phase average of multiple gaits following Winter et al. [27], and quantifies the variability of a parameter considering multiple subjects conducting the same physical activity. To compare the CVs of the knee angle and the hydrodynamic pressure, the *z-scored Coefficient of Variation* (CV_z_) was calculated. This was done for each time-stamp over a gait cycle, where the z-scored values were obtained by subtracting the time stamp mean and dividing by the time stamp standard deviation. The equations and descriptions of statistical methods applied for the validation of the devices and the cross-comparison of land and water gait parameters are provided in the supporting information, S2 Appendix.

## Results

The temporal gait parameters, knee angle and lateral hydrodynamic pressure were calculated on a total of 159 samples, as one subject had only 9 acceptable trials. Data were tested for normality using a Kolmogorov-Smirnov test, and were found to be non-normally distributed for the majority of trials. Accordingly, median and interquartile ranges (IQR) were used as summary statistics for further comparison. Table 2 summarizes for the optoelectronic system (land only) and for the PIMU land and water trials the results for the temporal and knee angle parameters, as well as the CV and CV_z_ of the knee angle.

**Table 2:** Temporal gait and knee joint parameters estimated from the optoelectronic land trials and PIMU system for land and water trials. The summary statistics for comparison include the median, first (Q1), third (Q3) quartiles and interquartile ranges (IQR) of the distributions, as well as the knee joint coefficients of variation (CV and CV_z_).

		Optoelectronic		PIMU Land		PIMU Water	
		Median (Q1, Q3)	IQR	Median (Q1, Q3)	IQR	Median (Q1, Q3)	IQR
Temporal gait parameters	Stride time [s]	1.12 (1.04, 1.21)	0.17	1.11 (1.04, 1.21)	0.17	3.02 (2.61, 3.33)	0.72
	Stance time [s]	0.70 (0.64, 0.77)	0.13	0.66 (0.62, 0.71)	0.09	1.73 (1.54, 2.04)	0.50
	Swing time [s]	0.41 (0.40, 0.45)	0.05	0.46 (0.41, 0.51)	0.10	1.23 (1.05, 1.43)	0.38
	Stance phase [%]	62.50 (61.57, 63.62)	2.05	58.59 (56.59, 60.72)	4.13	58.18 (56.08, 61.30)	5.22
	Swing phase [%]	37.50 (36.38, 38.43)	2.05	41.41 (39.28, 43.41)	4.13	41.82 (38.70, 43.92)	5.22
Knee joint parameters	ROM [°]	62.70 (58.53, 67.33)	8.80	60.79 (57.41, 65.56)	8.15	60.19 (50.00, 72.01)	22.01
	Max flexion [°]	66.44 (61.32, 69.01)	7.69	63.93 (59.46, 69.39)	9.93	65.71 (54.97, 78.02)	23.05
	Max extension [°]	1.76 (-2.16, 5.40)	7.56	2.34 (0.45, 5.22)	4.77	6.37 (0.99, 11.85)	10.86
	CV [%]	27.84		31.02		60.57	
	CV_z_ [%]	20.97		31.20		67.69	

### Validation of the technology

The proposed PIMU technology was confronted against the optoelectronic system during the land trial to assess its reliability. It was found that the median and IQR of stride time estimations were consistent between optoelectronic and PIMU measurements (Table 2). Differently, the PIMU underestimated the stance time when compared to the optoelectronic system (median difference of 4%), and therefore overestimated the swing time by the same percentage. The validation parameters used to compare the optoelectronic and PIMU data for land-based trials are reported in Table 3. Considering temporal gait parameters, the results show a modest RMSE difference ranging from 0.03 to 0.06s, and acceptable values of the Spearman correlation coefficient with the lowest value of 0.80 for the swing time, 0.84 for the stance time and a maximum of 0.95 for the stride time. The Bland-Altman measurement biases (averages of the differences) for stride and stance times are minimal, suggesting a slight underestimation of the parameter by the IMU method. In the supporting information S1 Fig. are provided additional Bland-Altman plots of the gait temporal parameters. The Brunner-Munzel test was applied to evaluate differences between the temporal gait parameters obtained from the optoelectronic and the PIMU methods. Stride times did not differ significantly between methods (p = 0.60) whereas both the stance and swing times were found to be significantly different (p<0.001).

**Table 3:** Validation parameters used to compare the optoelectronic and the PIMU system for land-based trials. Root Mean Squared Error (RMSE), Spearman correlation coefficient (*ρ*), Bland-Altman plot coefficients: mean of the differences (bias), the standard deviation of the differences (*σ*), lower and upper boundaries of the confidence interval (CI = bias ± 1.96*σ*).

	RMSE	*ρ*	Bland-Altman plot coefficients			
			bias	*σ*	CI low	CI up
Stride time [s]	0.03	0.95	0.01	0.03	-0.06	0.07
Stance time [s]	0.06	0.84	0.05	0.04	-0.03	0.12
Swing time [s]	0.05	0.80	-0.04	0.04	-0.11	0.03
Knee angle [°]	10.96	0.74	-2.94	10.56	-23.64	17.76
Knee ROM [°]	3.81	0.81	1.40	3.55	-5.56	8.36

**Fig 2:**
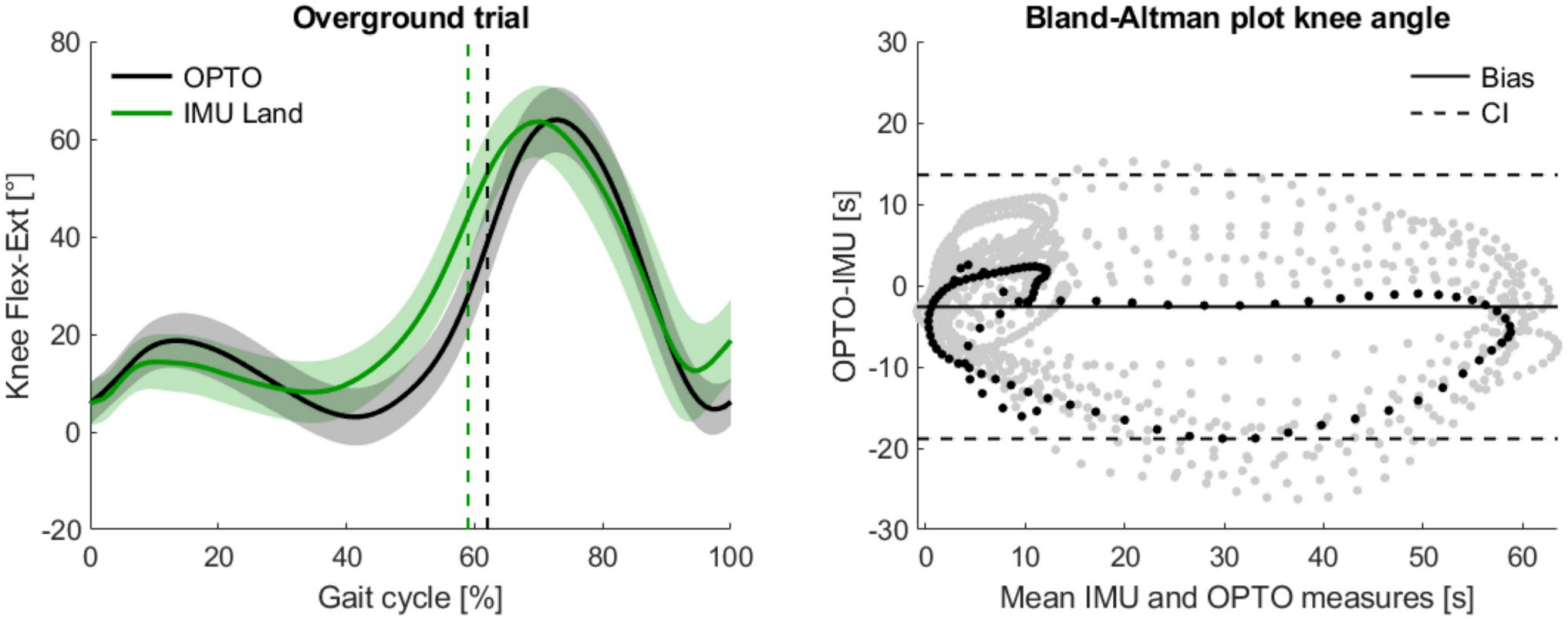
Comparison of optoelectronic and PIMU systems estimation of the knee angle for land-based trials. (Left) Mean and standard deviation of the knee joint flexion-extension angle, normalized over the gait cycle. Dashed vertical lines identify the toe-off. (Right) Bland-Altman plot of a single test subject with 10 repetitions (gray dots). A single gait cycle is highlighted (black points), and the bias (solid black line) and upper and lower 95% confidence interval boundaries (dashed gray lines) indicate that there were few substantial deviations between optoelectronic and PIMU knee angle estimates.

Regarding the knee joint parameters (Table 2) it was observed that the PIMU underestimated the ROM by 1.91° and the maximal flexion by 2.51°, expressed as the differences between medians. The CV comparison also revealed that PIMU knee joint variability on land was slightly higher, at 31.02%, compared to the 27.84% of the optoelectronic system. The CV values for both systems on land are in good agreement with the reference inter-subject CV for slow cadence of 26%, as defined by Winter and colleagues [35]. The knee joint validation parameters in Table 3 report a RMSE of 10.96° when comparing PIMU and optoelectronic measurements over the normalized gait. The Bland-Altman bias of less than 3° and a RMSE of the ROM of 3.81° indicate a small measurement bias of the PIMU and confirm the overall reliability of the proposed PIMU system to assess knee joint parameters during a walking gait. The knee flexion-extension angle results for the all land-based trials comparing the optoelectronic and PIMU systems are provided in the left panel of Fig 2. A Bland-Altman plot for the knee angle estimation of a single test subject is shown in the right panel of Fig 2. In general, the ROM estimated by the IMU sensors was found to be marginally lower (1.40°) when compared to the optoelectronic system on land. The PIMU knee flexion angle also exhibited a reduced peak value during the stance phase (0 to 20% of the stride) and was found to have a higher residual flexion angle at the termination of the gait cycle being around 18°, while about 6° for the optolectronic system. Statistical differences between optoelectronic and PIMU observations were evaluated using the Brunner-Munzel test, where it was found that knee ROM (p = 0.02) and maximal extension (p = 0.02) were significantly different and the maximal flexion was not (p = 0.67).

### Gait differences on land and underwater

**Fig 3:**
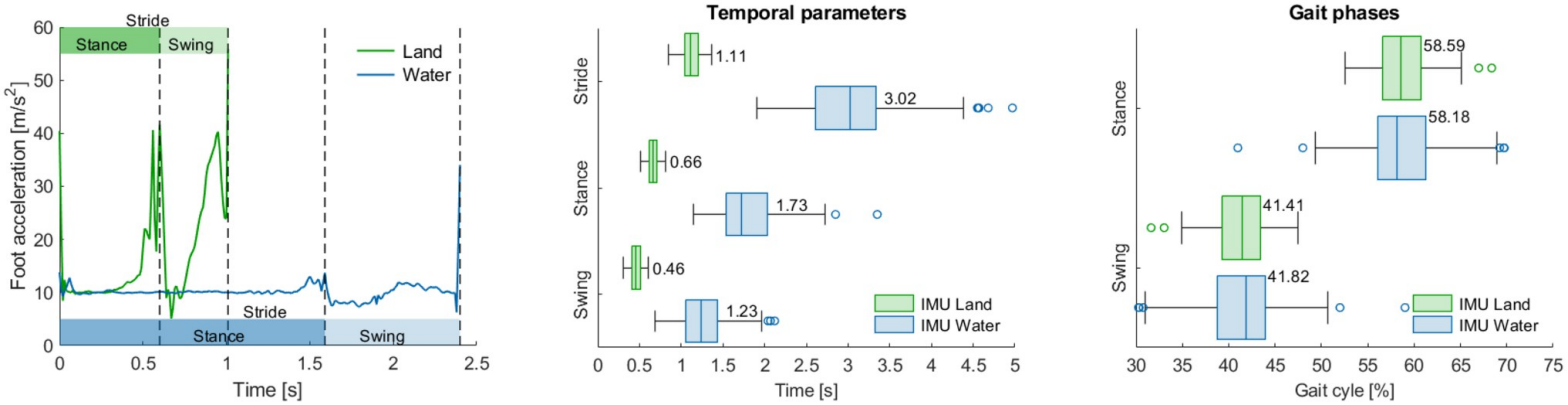
Comparison of the temporal gait parameters between land and underwater trials. (Left) Foot-mounted accelerometer magnitude time series during a single gait cycle on land and in water. The stride time of the underwater gait is nearly 2.5x longer in duration than that of the stride time on land. (Center) Boxplots of the temporal gait parameters. (Right) Boxplots of the gait phases. Boxes represent the interquartile range (IQR) over the 25th to 75th percentiles, the centerline corresponds to the median value of the distribution, error bars extend by a factor of 1.5 from the IQR and outliers are marked as circles.

The kinematic differences between the walking gait on land and in water from healthy test subjects were investigated comparing temporal and knee joint estimations of the two trials estimated with the PIMUs. Temporal gait events (heel strike and toe-off) were identified using peaks in the acceleration magnitude obtained from the foot-mounted PIMU for all trials, an example is shown in the leftmost panel of Fig 3. Boxplots of the estimated temporal gait parameters and gait phases are provided in the center and right panels of Fig 3, respectively. All the parameters reported increased variability during the water trial (Table 2). Compared to the parameters of the land-based trials, the underwater stride duration increased by a factor of 172%, while it was observed that the stance time increased by 162% and the swing phase by 167%. Despite the substantial increase in the temporal gait parameters observed in water, the subdivision into stance and swing phases remained similar to the overground assessments, respectively about 58% and 42% of the gait cycle (rightmost panel of Fig 3).

**Fig 4:**
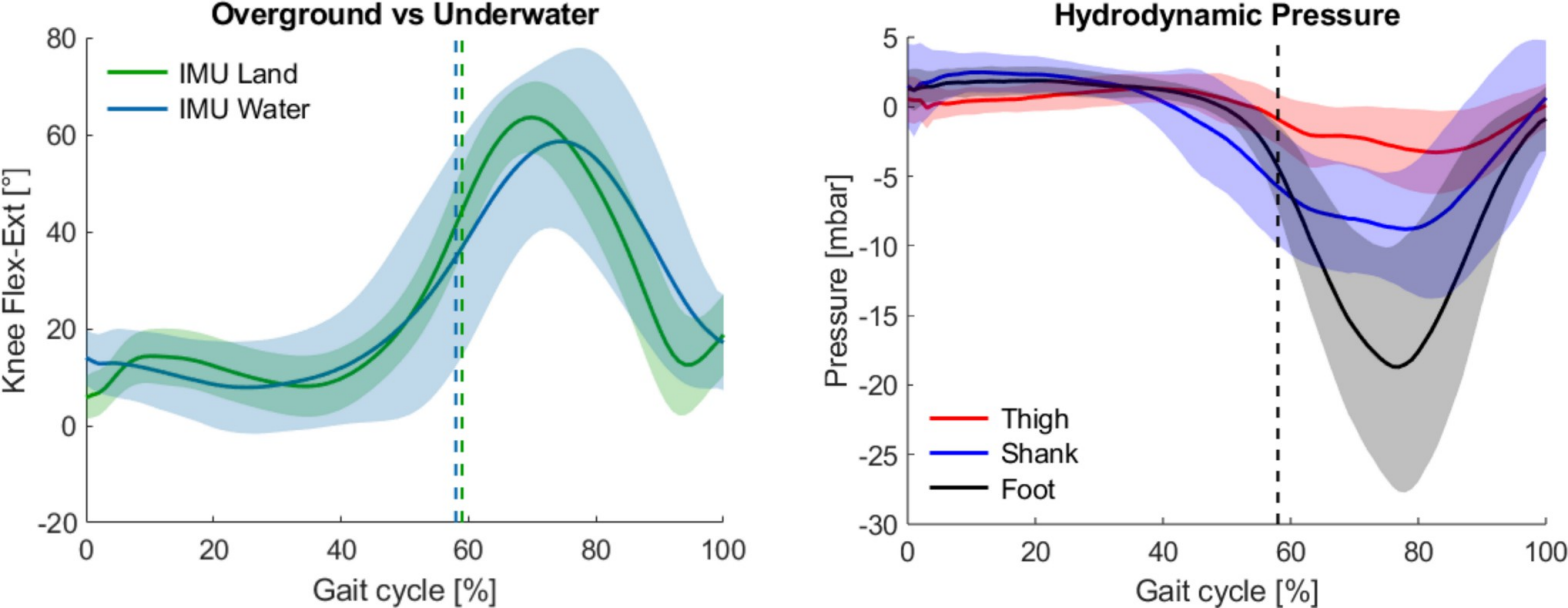
Normalized knee flexion-extension angles and hydrodynamic pressure over the gait cycle. (Left) Mean and standard deviation envelope (shaded regions) of the knee joint angle calculated from land (green) and water (blue) trials with PIMUs. (Right) Hydrodynamic pressure envelopes (shaded regions) obtained from the underwater trials from the PIMU mounted on the thigh (red), shank (blue) and foot (black). Dashed vertical lines in both panels indicate the toe-off.

The differences between land and underwater knee flexion-extension angles were modest, as shown in the left panel of Fig 4. Overall, the underwater gait reported increased variability, as indicated by the IQR of the knee angle parameters and a near doubling of the CV values when compared with land-based trials (Table 2). Interestingly, the knee angle in water did not exhibit an initial peak during the stance phase, where subjects showed an average 18° knee angle at 0% of the gait cycle. In addition, while it was found that the median values of maximal flexion and extension were higher in water of respectively 1.78° and 4.03°, a similar ROM was observed between the two environments. The underwater and overground gait and joint parameters were found to have significant differences (p<0.001) based on the Brunner-Munzel test, with the exception of the knee range of motion (p = 0.46) and knee maximal flexion (p = 0.47).

### Lateral hydrodynamic pressure

During the underwater trial, the hydrodynamic pressure was recorded and normalized over the gait cycle. The mean pressure over the gait and standard deviation envelopes are shown in the right panel of Fig 4 for the thigh, shank and foot PIMU locations. Characteristic behaviours can be observed for each of the three sensor mounting locations. During the majority of the stance phase, up to about 40% of the gait cycle, the three signals remained close to their initial pressure values. The pressure on the shank begins to decrease at 40% of the gait cycle, indicating that the leg is lifting towards the water surface, whereas the thigh and foot pressures remained nearly stationary until 50% of the gait. This portion of the gait cycle corresponds to the heel-off, where the foot begins to leave the ground and prepare for the swing phase. After the 50% of the gait, the thigh and foot pressure were observed to decrease, across the pre-swing and initial swing phases (50 to 80% of the gait). Finally, for the last portion of the gait cycle (80% to 100%), during the mid and terminal swing phases, the leg prepares for the following heel contact and the hydrodynamic pressure continues to increase and until it reaches its initial value as the leg returns back to the floor. The hydrodynamic pressure fluctuation amplitude was observed to be proportional to the amount of movement: smaller for the thigh, intermediate for the shank and larger for the foot. Moreover, the thigh and shank average pressure showed two distinct curves at about the 60% and 80% of the gait, corresponding to the initial swing and mid-swing phases.

To quantify the behaviour of the hydrodynamic pressure during underwater gait, parameters analogous to the ones estimated for the knee angle have been approximated and are reported in Table 4. Among the three locations, the pressure measured on the thigh showed the smallest ROP both in terms of median (7.68mbar) and IQR (5.60mbar), as well as the biggest CV (138.35%). Conversely, the foot location reported the biggest ROP (19.97mbar with an IQR of 11.78mbar) and the smallest CV (80.06%). Finally, the shank location displayed intermediate values for both ROP (13.98 with an IQR of 8.34) and CV (98.99%). The normalization of the CV (CV_z_ in Table 4) confirms that the hydrodynamic pressure on the foot (CV_z_ = 39.65%) is more reproducible between subjects and repetitions.

**Table 4:** Hydrodynamic pressure statistics of underwater gaits. Sensors were placed on the thigh, shank and foot and observations were summarized as the median, first (Q1), third (Q3) quartiles and interquartile ranges (IQR) of the Range of Pressure (ROP), maximal and minimal pressure, as well as the coefficient of variation (CV and CV_z_).

	Thigh		Shank		Foot	
	Median (Q1, Q3)	IQR	Median (Q1, Q3)	IQR	Median (Q1, Q3)	IQR
ROP [mbar]	7.68 (4.20, 9.84)	5.64	13.98 (11.04, 19.52)	8.48	19.97 (16.69, 28.69)	12.00
Max pressure [mbar]	2.50 (1.66, 3.30)	1.64	3.13 (2.31, 4.10)	1.79	2.43 (1.79, 3.27)	1.48
Min pressure [mbar]	-4.54 (-7.07, -2.51)	4.56	-11.35 (-15.79, -7.80)	7.99	-17.64 (-26.79, -14.12)	12.67
CV [%]	138.34		98.99		80.06	
CV_z_ [%]	126.88		70.15		39.65	

## Discussion

Previous studies [13, 16] have demonstrated the potential of IMU-based systems for underwater quantitative motion analysis. However, a recent systematic review by the authors [5] revealed a lack of suitable wearable IMU-based technologies and parameters for monitoring physical activity in water. To address these gaps, this work developed novel wearable devices that combine IMU and pressure sensors suitable for both overground and underwater gait analysis. Hydrodynamic pressure was proposed as a unique parameter that reflects the interaction between the body and the fluid during motion. The devices were tested on 16 healthy adults who performed gait analysis in both overground and underwater conditions. A total of 159 trials were collected and analyzed per condition for temporal and joint gait parameters.

### Validation of the technology

The proposed PIMU sensors were validated by comparing their performance with an optoelectronic system in a motion analysis laboratory. The temporal gait parameters measured by the PIMU sensors had a low RMSE (up to 5% of the gait cycle for stance time) and a high Spearman coefficient (above 0.8), which are consistent with similar studies [36]. The Bland-Altman bias was also small, especially for stride time [37]. The knee joint estimation by the PIMU sensors reported had a relatively high RMSE with respect to the optoelectronic assessments for both the whole curves and ROM, but a low Bland Altman bias (around -3°), which is aligned with [38]. The Brunner-Munzel test reported statistically significant differences between optoelectronic and PIMU gait parameters, with the exception of the stride time and maximal flexion angles, which were found to be highly comparable. Moreover, the algorithm used in this study to estimate the knee flexion-extension [31] improved the RMSE and CV of the knee joint estimation compared to the previous algorithm used by the authors in a similar investigation [18], and achieved a similar CV as the optoelectronic system (31.02% vs 27.84%), as shown in Table 2.

These results indicate that the PIMU sensors have good accuracy and reliability for motion analysis in water, and can measure both temporal and joint parameters with a reasonable trade-off between precision and ease of application.

### Differences between the two environments

When applied to the water environment, PIMUs registered temporal and kinematic parameters in good agreement with previous IMU-based investigations [15–17]. The results of this study indicate the gait cycle duration more than doubled in water, while the proportion of stance and swing phases was constant in the two environments, comparably to previous researches [39]. However, a larger variability, indicated by the interquartile range, has been observed in the temporal parameters (Table 2). A similar trend was found for the knee joint angle assessment, where values of ROM were comparable to previous studies with IQR doubled in water for all the joint parameters [40]. Additionally, the CV of the knee joint in water more than doubled when compared to the land-based trials. Therefore, we conclude that underwater gait kinematics are likely to be inherently less repeatable than those on land. Potentially caused by the kinematic differences observed between underwater and overground kinematics (Fig 3), was further confirmed by the Brunner-Munzel test results of the gait parameters, which indicated statistically significant differences between walking gaits on land and underwater. For example, the knee flexion curve at heel strike and during the load acceptance phase was absent in water [39]. This could be related to the slower walking speed in water, which has been shown to reduce the flexion angle during initial stance [10]. Moreover, the maximal flexion angle during the swing phase in water was smaller than on land. This finding is consistent with some studies [10, 15], but not with others [39]. Previous works have pointed out that such discrepancies may be partially explained by different experimental conditions, such as water depth and participants’ age [15]. To account for the effect of participants’ height on the indexes estimated, we also calculated the CV for the knee angle after z-score normalization and found that this also is more than doubled in water.

These kinematic differences reflect the properties and resistance of the fluid medium. Water density imposes higher resistance to movement, which can lead to slower, less controlled and less repeatable motions and different motor strategies [15]. These factors limit the cross-comparison of results between subjects and studies on land and underwater.

### Lateral hydrodynamic pressure

The higher density of water provides substantially more resistance to the movement than air resulting in various advantages in terms of rehabilitation and wellness. In this study, a characterization of the hydrodynamic pressure occurring on the lower limb during gait in water was performed. It is the first investigation attempting to describe the interaction between water and the body in motion exploiting the unique characteristics of water rather on relying only on inertial measurements. Three PIMU sensors were attached laterally on the thigh, shank and foot. The pressure data were processed as the knee angle data: data have been z-scored normalized to account for different heights and the ROP, as well as other parameters have been estimated (Table 4). It was found that the pressure on the thigh and shank was smaller, whereas the pressure on the foot was higher and more consistent, reporting a CV_z_ of 39.65%. Since the hydrodynamic pressure depends on the depth and to allow for a comparison with the knee angle variability, the coefficient of variation of both pressure and joint angle was estimated on z-scored data. The CV_z_ of the foot pressure was therefore compared with the CV_z_ of the knee angle on land (31.20%) and in water (67.69%). These results suggest that the foot hydrodynamic pressure may provide a less variable parameter to evaluate the walking gait underwater than the knee angle.

### Limitations of the study

The hydrodynamic pressure on the lower limbs has been proposed as a novel parameter for underwater gait analysis allowing a reasonable reconstruction of the gait phases. Nevertheless, the conducted study has severe limitations in the subjects and protocol considered.

Despite gathering reference data on a segment of the population, valuable for future investigations, this research involved only healthy adults. This does not allow for a comprehensive understanding of the importance of aquatic physical therapy and of the overall effects of the water on the body systems. The protocol also involved as physical activity of interest simple locomotion because of its relevance [6] and its well-known characteristics and established parameters [24]. However, other exercises commonly used in aquatic physical therapy [5] might help characterizing better the fluid-body interaction through the assessment of hydrodynamic pressure. Furthermore, this investigation exploited three pressure and inertial sensors positioned laterally to the leg, but it remains to be established if there are more suitable locations for the assessment of the hydrodynamic pressure. Future studies should therefore investigate different sensor positions and alternative tasks, as well as consider different populations, to improve our fundamental knowledge of water’s effects on lower limb kinematics. The use of IMUs resulted in the investigation of solely the flexion-extension angle limiting the analysis of the knee joint kinematics to the sagittal plane and to its superficial angle. This, limited the study of underwater locomotion and might be especially restricting when considering pathological subjects, for which the analysis of all the anatomical planes is fundamental [41]. The interpretation of the results is somewhat dependent on the particular choice of statistical method used to test for significant differences between the sample populations. While being robust and effective, the Brunner-Munzel test as applied in this work cannot account for inter-group repeated measures. Accordingly, the authors cannot evaluate the differences between individuals using the same method and in the same environment. This assumption was tested in a previous work and determined to be acceptable when relying on ensemble statistics for activity recognition [42].

Additionally, IMUs which include magnetometers and rate gyroscopes are susceptible to ferromagnetic disturbances and gyroscopic drift that limited the synchronization ability of the three PIMUs, resulting in time-consuming data post-processing which affected the final estimation of kinematics parameters. Future improvements in the application of more advanced calibration algorithms [43, 44] and through the combination of IMU-based and computer vision assessments [18] are recommended to improve the efficiency and quality of PIMU-based gait analyses.

## Conclusions

This work investigated how wearable devices outfitted with IMU and pressure sensors can be used to improve the investigation of underwater motion analysis. In contrast to previous studies that relied on methods and parameters designed for land-based assessments [5], this work proposes a parameter tailored to the underwater environment. The devices have been initially validated through a performance comparison against the gold standard optoelectronic system focusing on temporal and knee joint parameters during gait. The results indicate acceptable accuracy and reliability. By applying the sensors to the aquatic environment, kinematic differences between overground and underwater gait and knee joint parameters have been observed pointing out that underwater locomotion is subjected to higher variability and uncertainty. For example, the stride time on land reported an IQR of 0.17s, while underwater of 0.72s, being respectively about 15% and 24% of the gait cycle. Similar conclusion can be reached considering the knee angle coefficient of variation that, after normalization (CV_z_), was 67.69% in water, a result far from the standard CV defined by Winter [35] of 26%. The variability of standard gait parameters in water lead us to the consideration of a new and alternative parameter: the hydrodynamic pressure exerted on the lower limbs during the gait cycle. The hydrodyanamic pressure was investigated for three lower limb locations, namely thigh, shank and foot, and was compared to the more commonly used knee flexion-extension angle. The normalized coefficient of variation on the foot was the lowest, 39.65%, suggesting that hydrodynamic pressure can provide a new parameter to complement traditional gait analysis. We propose the pressure as a measure of the interaction between the body in motion and the underwater environment to promote a comprehensive understanding of how the presence of the water changes the way we move in it.

## Supporting information

S1 Appendix Computation of temporal gait parameters. (TEX)

S2 Appendix Statistical indexes estimated throughout the investigation. (TEX)

S1 Fig Bland-Altman plots of the temporal parameters for the IMU validation. (ZIP)
